# Amplification of agriculture factor productivity, food price and exchange rate on societal welfare spiraling in Ethiopia

**DOI:** 10.1016/j.heliyon.2022.e10675

**Published:** 2022-09-17

**Authors:** Ferede Mengistie Alemu

**Affiliations:** College of Business and Economics, Department of Economics, Debre Tabor, Ethiopia

**Keywords:** Welfare, Agriculture productivity, Food price, ARDL and Ethiopia

## Abstract

The issue of welfare is a widespread issue that any country in the world would love to achieve. However, in Ethiopia, many societies are living in poverty because of high food prices emanating from fragile agricultural productivity and exchange rate devaluation. The aim of this study is to investigate the effects of agricultural factors productivity, food prices, and exchange rates on household welfare in Ethiopia. Based on the stochastic process of the variables, the autoregressive distributed lag model has been employed. The result of the model revealed that agricultural land productivity in the introduction episode depresses welfare and, latterly, it optimistically improves welfare. Nevertheless, labor productivity in agriculture has a negative impact on welfare. Furthermore, exchange rate depreciation and food price increases in Ethiopia endanger welfare by eroding purchasers' purchasing power and amplifying the divergence of demand and supply in the economy. To improve the welfare of society, the government and society should increase the productive capacity of domestic firms and the agriculture sector to the extent that offsets the exchange rate effects on welfare.

## 1 Introduction

Currently, the region of Sub-Saharan Africa (SSA) is home to about 224 million malnourished people. It accounts for about 25 percent of the world’s malnourished population. In 2015, 20.8 percent of the population was hungry, a figure that has risen dramatically to 22.7 percent in 2016 (Teka and Lee, 2020).

Agriculture in Ethiopia contributes 27.5 billion dollars, or 34.1% of GDP, employs 79% of the workforce, accounts for 79% of foreign earnings, and is the primary source of raw materials and capital for investment and market (Diriba, 2020).

There are two conflicting results about the impact of agricultural factors productivity, food prices, and exchange rates on societal welfare. Food security is a dynamic interaction of food environments, food acquisition and preparation preferences, and inspired agency (Wertheim-heck and Raneri, 2020). It confirmed that harmful diets in underprivileged countries replicated the types of diets expected in countries experiencing the nourishment changeover (Holdsworth et al., 2020). The welfare of households is deterministically affected by food price shocks, technological advancement, factor productivity, and animal production in the agriculture sector (Enahoro et al., 2019; Frija et al., 2020; Hill and Porter, 2017).

Moreover, the acquisition of large-scale agricultural land has a substantial welfare implication for the affected rural population (Kleemann and Thiele, 2014). Crop productivity can be amplified by market channels. Improving the commercial prospects of crops requires the appropriate institutional and policy interventions to facilitate innovation in production and marketing that makes high value products and reduces price volatility (Bekkers et al., 2017; Ikuemonisan and Akinbola, 2019; Petsakos et al., 2019). Welfare is indomitable via exchange-rate stability, which is often prospected as sympathetic to trade and to enhancing welfare (Bacchetta and Wincoop, 2000). The instantaneous reaction of labor’s income share to a one-standard deviation shock in exchange rate volatility is depressing (Goodness, 2019; Mekonnen, 2017; Sims and Wolff, 2018). Welfare was optimistically connected with the firm’s profit, and it was depressingly associated with regulatory capability pending all of the firms' hold to environmental law (Lei et al., 2017; Matita et al., 2021; Vidal et al., 2018). Empirical results in Ethiopia revealed that agriculture productivity that is constrained by climate change affects welfare negatively (Berhane et al., 2021; Eshete et al., 2020). Furthermore, the welfare of households in Ethiopia is challenged by climate change and price volatility that derive from the formulation of weak agricultural strategies and packages (Addisu, 2020). However, research findings in Ethiopia have not considered the exchange rate volatility on the welfare of society; rather, they consider the effects of agriculture productivity and price volatility on household welfare. They failed to consider the role of livestock production. Hence, the study aims to examine how exchange rate volatility affects the welfare of society. The general objective of the study is to examine the welfare effects of food prices, the exchange rate, and agriculture productivity in Ethiopia.

## 2 Literature

The issue of exchange rates is the translation of the currency of one country into the currency of another country. Exchange rate volatility understood as a computation of wavering can affect disparity through its impact on different economic variables, which in turn manipulate disparity. As a result, it has a variety of effects on the well-being of domestic citizens, in addition to serving as a standard for demonstrating the competitiveness of domestic industries in the global market (Goodness, 2019; Nguse et al., 2021; Suleiman et al., 2018).

Depending on preferences and the monetary-policy rules followed by each system, either exchange-rate system can be superior in terms of trade and welfare (Bacchetta and Wincoop, 2000). It is indispensable that we make a distinction that ought to be essential to designing policy, which in turn can accelerate social welfare via increasing market competitiveness. It is evidence that factor markets are misplaced in their entirety. The appropriate step is likely to be to create markets by assigning property rights and removing restrictions on certain forms of exchange (Dillon and Barrett, 2017). So offering that prior to a long-run rate, the exchange rate in reaction to an economic tremble in macroeconomic aggregates, at the outset, explodes beyond the new level to which it ultimately relaxes (Umoru and State, 2012). To make the process of trade logical and ensure the mutual benefit of countries, it is required to formulate favorable economic environments like exchange rate immovability and sympathetic policies such as banking and insurance. It is also essential in order to achieve everlasting production and business investment that enables us to accelerate the level of firms' profitability by dropping out of the business cycle (Feizi et al., 2021).

Urban households are less deprived than rural households. Poverty and welfare derivation are correlated and have been inversely determined by a household's education level, which enables them to look for new modes of income-generating activities. It has long been established that investing in rural dwellers' education and economic conditions is the primary tool for alleviating household poverty (Biyase and Zwane, 2018). There is a reasonable case to be made that there is an indirect co-integration of shock and agriculture, which tends to deprive the welfare of poor households (Nkang, 2018; Trigo and Cap, 2003). Uplifting agriculture productivity depends on espousing production-enhancing technologies and the innovativeness of participants in the sector, particularly farmers (Akudugu et al., 2012; Anang et al., 2020; Biru et al., 2020; Defar et al., 2017; Wong et al., 2020; Workineh et al., 2020).

Agricultural technology adoption positively affects the welfare of households in Ethiopia. Technologies could directly affect farm output, which translates into consumption at a household level. This also implies that the opportunity for enhancing the role of adoption of agricultural technologies is larger than what contributes to poverty reduction (Amare et al., 2014; Mekonnen, 2017; Mulugeta and Bekele, 2012).

It is concluded that the recurrent episodes of spikes in food prices raised poverty significantly, especially in urban areas of food-importing countries. High prices can also contribute to political unrest, and recent episodes have prompted many countries to enact protectionist policies (Anderson et al., 2019). Policy measures are essentially exaggerated price shocks. Protectionist measures by NGOs are inconsistent with their previous calls for the abolition of food production subsidies in rich countries to help farmers in poor countries (Moreno and Hector, 2012). Households are tremendously affected by changes in food prices, but their outcome varies depending on the income of households (Adekunle et al., 2020; Cedrez et al., 2020; Choga and Giwa, 2020; Quentin et al., 2015).

According to Wossen et al. (2018) High risk is a hindrance to farmers and thus, it has a negative association with food production as farmers are likely to shift investments from risk-prone to production of other non-agricultural products with less risk. It thus affects the welfare of the troubled producers and net consumers.

## 3 Methodology

In this study, the time series data from (1980220) has been used. The data is sourced from worldwide open data sources, mainly obtained from Penn World Data Source (PWt, 2020) and the World Development Indicator (WDI, 2020). It is crucial that a fitting methodology for the time series be applied, through which one can verify unbiased and reliable estimates. A method of selection for time series analysis is undertaken based on the stationery test results. If all the variables are stationary I(0), ordinary least square (OLS) or vector autoregressive (VAR) models can provide unbiased estimates.

If all the variables are non-stationary, ARDL is appropriate to analyze the relationship (Nkoro and Uko, 2016; Pesaran, 2008 and Shrestha and Bhatta, 2018).

To select the optimal lag length included in the model, Akaike and Schwarz's criteria were important to identify the right model that leads to accurate prediction and guarantees a sustainable production system (Ongbali et al., 2018). In practice, this can also be denoted as follows:

According to Nkoro and Uko (2016), the ARDL *(p, q1, q2......qk) the* model specification is given in Eq. (1) bellow:-

(1) $$\Delta xt=\delta 0i+\overset{k}{\underset{i=1}{\sum }}\alpha i\Delta xt-1+\overset{k}{\underset{i=1}{\sum }}\alpha 2\Delta yt-1+\beta 1xt-1+\beta 2yt-1+v1t$$

(2) $$\Delta yt=\delta 0i+\overset{k}{\underset{i=1}{\sum }}\alpha i\Delta yt-1+\overset{k}{\underset{i=1}{\sum }}\alpha 2\Delta xt-1+\beta 1yt-1+\beta xtt-1+v1t$$

From Eq. (2), where *k* is the ARDL model maximum lag order and chosen by the user. The selected ARDL (*k*) As per Pesaran (2008) model long run equation specified as;

(3) $$\Delta yt=\delta 0+\overset{k}{\underset{i=1}{\sum }}\alpha 1x1t\;+\overset{k}{\underset{i=1}{\sum }}\alpha 2x2t+\overset{k}{\underset{i=1}{\sum }}\alpha 3x3t+\overset{k}{\underset{i=1}{\sum }}anxtn+v1t$$

From the above Eq. (3) Where, *xs* (*x1t*, *x2*, *x3t*, ………... *xnt*) are the explanatory or the long run forcing variables, *k* is the number of optimum lag order. In the above Eq. (3), *k* lag length ARDL model, if there is co-integration or long run relationships between variables’, the error correction model has been specified (Pesaran, 2008).

(4) $$ECT=\varepsilon t=yt-\overset{k}{\underset{i=1}{\sum }}\beta ixit-\varphi \Delta xt+xt-1$$

In the above Eq. (4), if there is co-integration between variables, the error correction model has been preferable. The *ECT* shows how much of the disequilibrium is being corrected, that is, the extent to which any disequilibrium in the preceding period is being adjusted in *yt*. A positive coefficient indicates a departure, while a negative coefficient indicates convergence (Nkoro and Uko, 2016).

## 4 Result and discussion

### 4.1 Descriptive analysis

To analysis the time series data, descriptive method of analysis in considered as essential tool to describe the trend and relations between variables over time.

**Figure 1:**
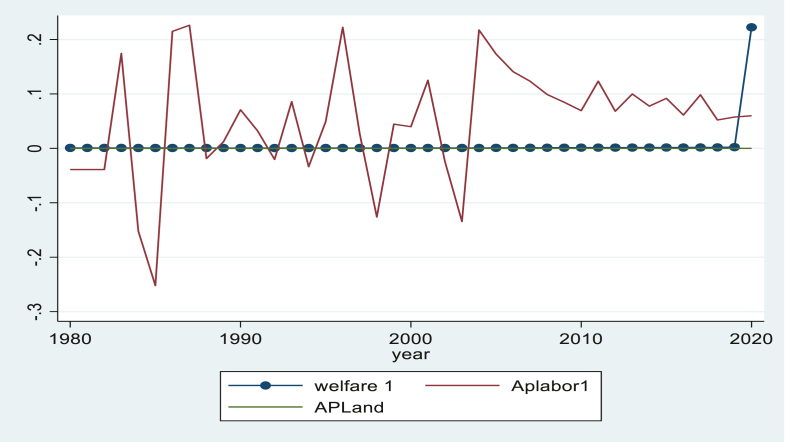
Welfare, agricultural land productivity and agricultural labor productivity in Ethiopia. *Source*:- own computation stata 16.

From the above Figure 1, agriculture labor productivity, which is measured by overall agriculture output per total labor employed in agriculture, has experienced some inconsistent alterations over time. From 1980 to 1985, agricultural labor productivity diminished radically, and it recovered from the downturn path until 1990. Laterally, it also reduced total labor productivity until 2000. It has not amusingly accelerated agricultural labor productivity in Ethiopia after 2005, indicating that agricultural labor productivity in Ethiopia has not made a constructive contribution to increasing societal consumption levels. The relationship between agricultural land productivity, which is determined as the total agriculture output per total arable land, and welfare in Ethiopia has shown some steady transformation over time. The total agricultural land productivity in Ethiopia from 1980 to 2020 revealed an inconsequential series that has confirmed the existence of stumpy input utilization, underuse of land, and squat average productivity of the land. Welfare in this study is defined as the total national consumption per total national population. As long as land and labor productivity in the agriculture sector increases at a constant rate, similar to per capita agriculture output, Ethiopian welfare will change at a steady rate.

**Figure 2:**
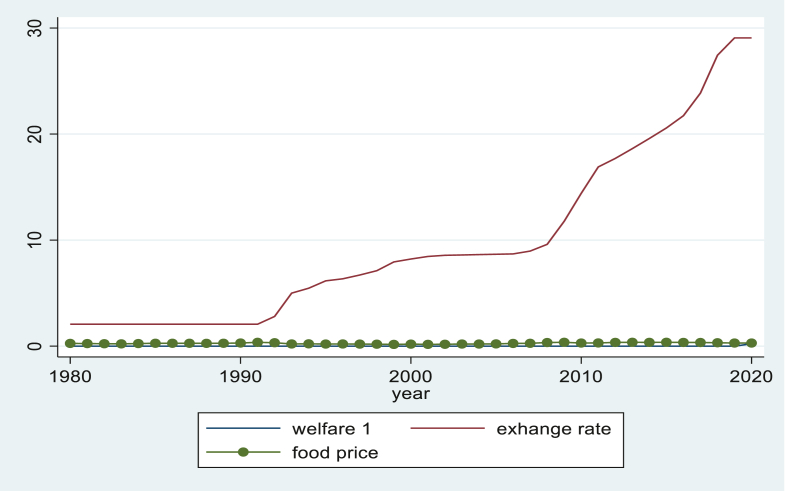
Welfare, Food price and Exchange rate in Ethiopia. *Source*:- own computation stata 16.

In the above Figure 2, in Ethiopia, the exchange rate has been changing at a constant rate since 1990, but subsequent to that, the exchange rate has been increasing at an increasing rate. This speedy devaluation of the exchange rate has a distractive upshot on the consumption level of households via a drop in the purchasing power of consumers and high food prices in Ethiopia. Food prices and household welfare in Ethiopia have most likely changed in proportion to the extent to which consumption levels have increased in response to an increase in food prices. In fact, exchange rates have adverse impacts on consumption levels, decreasing the purchasing power of consumers and limiting the volume of imports in favor of exports in developing countries. But it might have a positive contribution to enlarging welfare via increasing consumption, which it drives through high investment and remittance.

### 4.2 Econometrics analysis

If there is a long-run relationship between the underlying variables, it indicates the presence of co-integration between them. The matter of discovering the fitting lag length for each of the principal variables in the ARDL model is very imperative since it has Gaussian error terms (i.e., standard normal error terms that do not suffer from non-normality, autocorrelation, heteroskedasticity, etc.). In order to select the appropriate model for the long-run underlying equation, it is necessary to determine the optimum lag length(k) by using proper model order selection criteria such as the Akaike Information Criterion (AIC), Schwarz Bayesian Criterion (SBC), or Hannan-Quinn Criterion (HQC) (Nkoro and Uko, 2016). According to the methods, lag length selection results confirmed that the maximum lag of the underlining variables is 4. see the above Table 1.

**Table 1:** The optimal lag length selection.

Sample	Year 1985–2020				Number of observation = 36			
lag	LL	LR	df	p	FPE	AIC	HQIC	SBIC
0	564.266				1.4e−21	−31.0148	−30.9227	−30.7509
1	633.957	139.38	36	0.000	2.2e−22	−32.8865	−32.2417∗	−31.0391∗
2	678.419	88.924	36	0.000	1.6e−22∗	−33.3566	−32.1591	−29.9256
3	707.575	58.312	36	0.011	3.9e−22	−32.9764	−31.2262	−27.9619
4	760.396	105.64∗	36	0.000	5.3e−22	−33.9109∗	−31.608	−27.9619

*Source* - own computation stata 16.

Using ordinary least squares or other similar methods for non-stationary time series may produce spurious results. Hence, Engle and Granger (1987) developed a co-integration test method to analyze the relationships among non-stationary variables (Shrestha and Bhatta, 2018). Co integration involves a certain stationary linear combination of variables that are individually non-stationary but integrated to order, I(d) (Nkoro and Uko, 2016). From Table 2 results confirmed the absence of co integration and helped to use the ARDL model rather than the error correction model (ECM).

**Table 2:** Engle-Granger test for co-integration.

				N (1st step) = 40
				N (test) = 39
Test	Statistic Value	1% Critical	Critical 5%10%	10% Critical Value
Z(t)	−2.230	−5.940	−5.940	−5.940

The welfare of a society is the maximum consumption of output retrieved from the optimum employment of factors of production. The ARDL model result from Table 3 authenticated that the previous welfare, which is estimated by the total consumption per total population, is statistically and positively related to the current welfare of society in Ethiopia. It is statistically significant at a 1% level of significance. It is interpreted as when the previous annual total consumption per total population in Ethiopia increases, the current total consumption increases by 362.298 dollars. In fact, this positive affiliation between current and previous welfare can exist because the previous consumption level is a basis to achieve sustainable consumption in Ethiopia. Accelerating agricultural productivity via improving agricultural inputs like labor and land is a widely practiced package applied to resolve chronic poverty in developing countries. It is considered the most effective means of addressing poverty and the main pathway out of poverty. Increasing agricultural productivity helps to meet food security in countries with a rapidly growing population (Amare et al., 2014). The studies conducted by Mekonnen (2017) and Mulugeta and Bekele (2012) It is well established that agricultural productivity resulting from the adoption of technology has a direct contribution to welfare. Also, Trigo and Cap (2003), and (Akudugu et al., 2012) The improvement of technology in agriculture transmission holds the promise of enhancing the evolution of the sector from low-productivity subsistence agriculture to high-productivity agriculture.

**Table 3:** The Auto regressive distributed lag (ARDL) model results.

Sample: 1985–2020						Number of obs = 36
						F(12, 23) = 19.01
						Prob > F = 0.0000
						R-squared = 0.9084
						Adj R-squared = 0.8606
Log likelihood = 111.43343						Root MSE = 0.0137
Welfare	Coef.	Std.Err.	T	P > t	[95% Conf.	Interval]
welfare						
L1.	362.298	34.580	10.480	0.000	290.764	433.831∗∗∗
Agric- land productivity						

	66071.45	37727.88	1.750	0.093	−1.20e + 04	1.44e + 05∗
L1.	−−8.66e + 04	40518.28	−2.140	0.044	−1.70e + 05	−2748.325∗∗
L2.	−1.03e + 04	4280.603	−2.400	0.025	−1.91e + 04	−1398.710∗∗
Agric-labor productivity						

	−0.574	0.265	−2.170	0.041	−1.122	−0.027∗∗
L1.	0.546	0.282	1.940	0.065	−0.038	1.129∗
Food price	−0.356	0.090	−3.970	0.001	−0.542	−0.171∗∗∗
Exchange rate						

	−0.010	0.005	−2.250	0.034	−0.020	−0.001∗∗
L1.	−0.016	0.005	−3.360	0.003	−0.026	−0.006∗∗∗
L2.	−0.023	0.005	4.560	0.000	0.012	0.033∗∗∗
L3.	−0.010	0.005	−2.240	0.035	−0.020	−0.001∗∗
Food export	−0.001	0.001	−1.160	0.258	−0.002	0.001
_cons	0.015	0.004	3.410	0.002	0.006	0.024∗∗∗

*Note:* ∗∗∗, ∗∗, ∗ are represents the level of significance 1%, 5% and 10% significant level, respectively.

In this study, agriculture productivity is represented by land and labor. Agricultural land productivity is premeditated by total agriculture output per total arable land. In the model, the two previous land productivity data sets are included under analysis. All these are statistically significant. The current land productivity is positively allied with the welfare of society in Ethiopia. But in the previous year, land productivity in Ethiopia had a negative and significant impact on the welfare of households. The justification that exists behind this empirical result is that most of the time, an increase in agricultural land productivity persuaded societies to reinvest the total output rather than consumption until their income reached its climax. Households in developing countries were not liberated from risk and uncertainty, and the intensity of production at the primary stage of the production period was most probably low. This led them to be net investors and net savers rather than more purchasers. Due to this fact, the preceding land yield is harmfully linked to welfare, and welfare turns out to be amplified subsequently to the uninterrupted enlargement in land productivity. On the other hand, agriculture productivity is denoted by the total agricultural output per hour of total employed labor in agriculture. The results showed that agriculture labor productivity in the current and preceding years was statistically significant at the 5% and 10% levels of significance, respectively. The current year's labor productivity in Ethiopia is depressingly linked to welfare, while the preceding agricultural labor productivity is optimistically related to the welfare of the society. It is noticeable that, with regular technology, the marginal productivity of labor in the agriculture sector is zero. Therefore, an increase in the total number of laborers on a fixed area of arable land has not contributed to increasing the total agricultural output. Even if successive employment of labor in agriculture becomes a cause for the predicament of disguised unemployment, perhaps it will augment agricultural productivity. Following this authentic circumstance, which most likely exists in agriculture, the untimely period of labor productivity has a constructive effect on welfare since the number of laborers in the preceding year is comparatively smaller than the amount of labor employed in agriculture. i.e., as the amount of labor employed in agriculture is relatively lower, its average productivity becomes high and it has a positive contribution to household welfare.

The empirical findings confirmed that increases in food prices have a variety of effects on household welfare in Ethiopia. The size of reimbursement payments depends on a number of factors, including the size of reimbursement payments, productivity spillovers on smallholders, employment opportunities for disposed farmers, and changes in food prices (Kleemann and Thiele, 2014). Indeed, innovation has a noteworthy effect on food price fluctuations and therefore on pragmatics in the noticeable diminution in the welfare cost of rural food price volatility (Ikuemonisan and Akinbola, 2019). Food price volatility in developing countries is verified as it is driven by a high divergence between demand and supply of food products (Bekkers et al., 2017). In line with the findings of the preceding scholars, the findings of this study validated the depressing effect of rising food prices on welfare. Food prices are inversely related to welfare and it is statistically significant. In Ethiopia, there is a lofty departure between demand and supply for food products. This divergence of demand and supply in Ethiopia happened due to low adoption of modern technology, weak agricultural institutions, and low agricultural factor productivity. However, as the population growth rate increases over time, so do food prices, which have a knock-on effect on household consumption by reducing consumer purchasing power.

According to Suleiman et al. (2018), Bacchetta and Wincoop (2000) (Goodness, 2019), and (Dillon and Barrett, 2017) Exchange rate volatility has an indirect impact on the welfare of society through a decrease in the volume of imports and strengthens the competitiveness of manufacturing industries for export. The results of the ADL model confirmed that both the current and prior exchange rates have a meandering impact on welfare. As the Ethiopian exchange rate devalued, the volume of imported goods decreased in favor of exports. In the short run, devaluation always makes the welfare of society worse off. But in the long run, there is a condition that makes welfare better off welfare by increasing gross domestic product via exports and investments.

## 5 Conclusion

Welfare is the augmentation of society’s harvest consumption that has been derived from the efficient exploitation of factors of production. Scholars confirmed that welfare is constrained by both economic and institutional restraints like production, innovation, social programs, and allotment of products and resources. The issue of welfare is a static economic phenomenon that highlights the mounting total output consumption. To achieve the welfare of society in Ethiopia, the dynamic economic aspect (GDP) growth rate needs to be first capitalized. Agriculture productivity is an indispensable apparatus to boosting the national output in Ethiopia by adopting modern technology like innovation in improved seeds, fertilizer, and livestock reproduction, agriculture services, and constructing market links between agriculture and industry. Results of this study convey that agriculture productivity is a pathway to enhance the welfare of society in Ethiopia by escalating labor productivity with the adoption of complementary inputs like improved seed extension services etc. Due to this fact, the marginal productivity of labor in the agriculture sector is negative throughout time. If agriculture operated under constant technology, its implications for the welfare of society would be negative. Land productivity is positively interconnected with the welfare of society, which means that when land productivity increases through the adoption of modern technology and the formulation of good agricultural institutions, it can improve the welfare of society in Ethiopia. Furthermore, the problem of food price increases and exchange rates in Ethiopia over the last 40 years has had a negative impact on consumption levels by increasing the disparities between food demand and supply and decreasing consumer purchasing power. Hence, the study recommended that the government of Ethiopia should reduce food prices and exchange rate volatility. Also, governments ought to be increasing the market share between industry and agriculture by establishing agricultural processing industries in order to increase food supply. The exchange rate should be evaluated by considering the net benefit of foreign investors investing in the industrial sector rather than agriculture investments.

## Declarations

### Author contribution statement

Ferede Mengistie Alemu: Conceived and designed the experiments; Performed the experiments; Analyzed and interpreted the data; Contributed reagents, materials, analysis tools or data; Wrote the paper.

### Funding statement

This research did not receive any specific grant from funding agencies in the public, commercial, or not-for-profit sectors.

### Data availability statement

Data associated with this study has been deposited at World Bank data source and Penn World Database under the accession number 5028.

### Declaration of interest’s statement

The authors declare no conflict of interest.

### Additional information

No additional information is available for this paper.
